# Development of an Australian cardiovascular disease mortality risk score using multiple imputation and recalibration from national statistics

**DOI:** 10.1186/s12872-016-0462-5

**Published:** 2017-01-06

**Authors:** Kathryn Backholer, Yoichiro Hirakawa, Andrew Tonkin, Graham Giles, Dianna J. Magliano, Stephen Colagiuri, Mark Harris, Paul Mitchell, Mark Nelson, Jonathan E. Shaw, David Simmons, Leon Simons, Anne Taylor, Jessica Harding, Bamini Gopinath, Mark Woodward

**Affiliations:** 1 Baker IDI Heart and Diabetes Institute, Melbourne, Australia; 2 Centre for Population Health, Deakin University, Melbourne, Australia; 3 School of Public Health and Preventive Medicine, Monash University, Melbourne, Australia; 4 The George Institute for Global Health, University of Sydney, Sydney, Australia; 5 Cancer Epidemiology Centre, Cancer Council Victoria, Melbourne, Australia; 6 The Boden Institute, University of Sydney, Sydney, Australia; 7 The Centre for Primary Health Care and Equity, University of New South Wales, Sydney, Australia; 8 Centre for Vision Research, Westmead Institute for Medical Research and University of Sydney, Westmead, Australia; 9 Menzies Institute for Medical Research, University of Tasmania, Hobart, Australia; 10 University of Melbourne, Melbourne, Australia; 11 Western Sydney University, Sydney, Australia; 12 UNSW Lipid Research Department, St Vincent’s Hospital, Sydney, Australia; 13 Population Research & Outcome Studies, The University of Adelaide, Adelaide, Australia; 14 The George Institute for Global Health, University of Oxford, Oxford, UK

**Keywords:** Cardiovascular disease, Risk assessment, Imputation, Recalibration

## Abstract

**Objective:**

To develop and recalibrate an Australian 5-year cardiovascular disease (CVD) mortality risk score to produce contemporary predictions of risk.

**Methods:**

Data were pooled from six Australian cohort studies (*n* = 54,829), with baseline data collected between 1989 and 2003. Participants included were aged 40–74 years and free of CVD at baseline. Variables were harmonised across studies and missing data were imputed using multiple imputation. Cox proportional hazards models were used to estimate the risk of CVD mortality associated with factors mutually independently predictive (p < 0.05) and a 5-year risk prediction algorithm was constructed. This algorithm was recalibrated to reflect contemporary national levels of CVD mortality and risk factors using national statistics.

**Results:**

Over a mean 16.6 years follow-up, 1375 participants in the six studies died from CVD. The prediction model included age, sex, smoking, diabetes, systolic blood pressure, total and high-density lipoprotein cholesterol (HDLC), a social deprivation score, estimated glomerular filtration rate and its square and interactions of sex with diabetes, HDLC and deprivation score, and of age with systolic blood pressure and smoking. This model discriminated well when applied to a Scottish study population (c-statistic (95% confidence interval): 0.751 (0.709, 0.793)). Recalibration generally increased estimated risks, but well below those predicted by the European SCORE models.

**Conclusions:**

The resulting risk score, which includes markers of both chronic kidney disease and socioeconomic deprivation, is the first CVD mortality risk prediction tool for Australia to be derived using Australian data. The primary model, and the method of recalibration, is applicable elsewhere.

**Electronic supplementary material:**

The online version of this article (doi:10.1186/s12872-016-0462-5) contains supplementary material, which is available to authorized users.

## Background

Australia has based its national cardiovascular disease (CVD) guidelines [1] on the Framingham risk score, which was developed using data from a small, middle-class, predominately White, population from a single town in the USA [2]. Data were accrued from 1948 and mostly cover a time when CVD incidence rates were relatively high and the rates of obesity and diabetes were relatively low. Moreover, the accuracy of the Framingham risk score is limited by the omission of important independent risk factors, including socioeconomic deprivation [3, 4] and markers of chronic kidney disease [5]. Thus, the suitability of the Framingham risk score for use in a contemporary Australian population is questionable.

The development of a CVD risk score in Australia has been hampered by the lack of a large Australian cohort study with information on all relevant risk factors and a sufficient number of CVD outcomes. We thus used combined data from the largest pool of Australian data available to us to develop an Australian 5-year CVD mortality risk score, which accounts for socio-demographic factors and markers chronic kidney disease, recalibrating the sample-based results using national statistics so as to produce contemporary predictions of risk.

## Methods

We considered all known Australian cohorts for inclusion in this study. An expert steering committee was established, which agreed, a priori, that the aim was to develop a 5-year CVD risk score using Australian data that included, unless there was evidence otherwise, measures of socioeconomic status, family history of CVD and markers for renal disease, in addition to the classical Framingham risk factors. The 5-year time frame was chosen for the following reasons: i) to reflect current absolute risk guidelines in Australia, which is based on 5-year risk of a CVD event [1], ii) focus group testing has shown Australian consumer preference for a shorter 5-year time frame over a 10-year time-frame for risk prediction [6] and iii) to enable modelling of treatment effects in RCTs, which are of a relatively shorter duration. Cohorts were included if they had data on CVD outcomes and on traditional CVD risk factors (age, sex, diabetes, systolic blood pressure (SBP), total cholesterol (TC), high-density lipoprotein cholesterol (HDLC) and smoking) and socioeconomic deprivation, measured by the Australian Socioeconomic Index For Areas (SEIFA) postcode-based score for some, or all, participants [7]. Cohorts were excluded if they were derived from a high-risk CVD population or if all participants were aged less than 40 years or older than 74 years. Cohorts were additionally excluded if information on prior CVD was unavailable. Six prospective cohorts, whose investigators were willing and able to contribute individual participant data, were subsequently identified (Table 1). Data were pooled and the relevant variables were harmonised across studies. These cohorts contributed to the Australian and New Zealand Diabetes and Cancer Collaboration [8]. This study was approved by the Alfred Health Human Research Ethics Committee (HREC; 310/14) and the Australian Institute for Health and Welfare (AIHW) HREC (2015/1/142).

**Table 1:** Summary data (mean and standard deviation or number (%)) and number (%) missing for putative risk factors, by study

	AusDiab		BMES		CUDS		Dubbo		MCCS		NWAHS		All Cohorts	
	Summary	Missing	Summary	Missing	Summary	Missing	Summary	Missing	Summary	Missing	Summary	Missing	Summary	Missing
n	7417		3558		894		1747		38897		2316		54829	
Age	54.06 (9.49)	nil	61.14 (6.91)	nil	55.00 (9.72)	nil	65.86 (4.3)	nil	55.02 (8.61)	nil	54.61 (9.77)	nil	55.62 (8.94)	nil
Women, n (%)	4103 (55)	nil	2063 (58)	nil	510 (57)	nil	987 (57)	nil	23430 (60)	nil	1241 (54)	nil	32582 (59)	nil
Systolic blood pressure	130.44 (18.19)	30 (0.4)	142.37 (19.94)	17 (0.48)	132.11 (21.70)	nil	144.17 (22.19)	nil	136.55 (19.0)	88 (0.23)	130.23 (17.86)	1 (0.04)	136.01 (19.34)	136 (0.25)
Total cholesterol	5.79 (1.05)	1 (0.01)	5.92 (1.05)	442 (12.42)	5.42 (0.98)	nil	6.57 (1.23)	4 (0.23)	5.53 (1.06)	148 (0.38)	5.45 (1.03)	17 (0.73)	5.61 (1.08)	612 (1.16)
HDL cholesterol	1.43 (0.39)	2 (0.03)	1.45 (0.43)	445 (12.51)	1.45 (0.39)	nil	1.38 (0.38)	7 (0.40)	1.41 (0.40)	33815 (86.93)	1.37 (0.39)	18 (0.72)	1.42 (0.40)	34286 (62.5)
Diabetes, n (%)	458 (6)	1 (0.01)	204 (6)	3 (0.08)	65 (7.3)	nil	85 (5)	nil	1478 (4)	6 (0.02)	142 (6)	nil	2432 (4)	10 (0.02)
FPG (mmol/L)	5.60 (1.22)	1 (0.01)	5.28 (1.50)	342 (9.10)	5.27 (1.50)	nil	5.32 (1.68)	11 (0.60)	5.65 (1.10)	12659 (32.5)*	5.40 (1.50)	nil	5.70 (1.51)	13013 (23.4)
SEIFA fifth, n (%)		17 (0.22)		6 (0.16)		6 (0.75)		nil		128 (0.33)		8 (0.32)		165 (0.30)
1st (most disadvantaged)	625 (8.43)		nil		558 (62.42)		1747 (100)		5537 (14.28)		843 (36.40)		9310 (16.98)	
2nd	1310 (17.66)		2466 (69.31)		237 (26.51)		nil		8023 (20.69)		537 (23.219)		12573 (22.93)	
3rd	2151 (29.00)		nil		93 (10.40)		nil		7149 (18.44)		371 (16.02)		9764 (17.781)	
4th	1460 (19.68)		1085 (30.49)		nil		nil		7978 (20.58)		473 (20.42)		10996 (20.06)	
5th (least disadvantaged)	1854 (25.00)		1 (0.03)		nil		nil		10082 (26.01)		84 (3.63)		12021 (21.92)	
Current smoker, n (%)	1050 (14)	131 (1.77)	558 (16)	116 (3.26)	137 (15)	nil	309 (18)	18 (1.03)	4378 (11)	10 (0.03)	453 (20)	16 (0.69)	6885 (13)	291 (0.53)
eGFR (ml/min/m^2^)	94.11 (14.04)	33 (0.44)	66.96 (14.78)	795 (22.34)	87.15 (15.73)	nil	n/a	1747 (100)	n/a	38897 (100)	n/a	2316 (100)	86.75 (18.46)	43788 (79.86)
Albumin-creatinine ratio	1.61 (7.22)	37 (0.50)	n/a	3558 (100)	n/a	894 (100)	n/a	1747 (100)	n/a	38897 (100)	n/a	2316 (100)	1.61 (7.22)	47449 (86.55)
Family history CVD, n (%)	n/a	7417 (100)	n/a	3558 (100)	n/a	894	603 (35)	nil	19830 (51)	nil	1551 (67)	34 (1.47)	21984 (40)	11903 (27.71)
BMI	27.26 (4.97)	64 (0.86)	26.99 (4.86)	40 (1.22)	28.18 (5.19)	2 (0.22)	26.12 (4.25)	2 (0.11)	26.86 (4.42)	27 (0.07)	28.50 (5.48)	1 (0.04)	26.99 (4.60)	136 (0.25)
High school +, n (%)	2692 (36)	4 (0.05)	2130 (60)	222 (6.24)	211 (24)	nil	490 (29)	42 (2.40)	12797 (33)	9 (0.02)	1204 (52)	656 (2.42)	19524 (36)	333 (0.61)
CVD death, n (%)	87 (1.17)	nil	246 (6.91)	nil	6 (0.67)	nil	320 (18.32)	nil	691 (1.80)	606 (1.56)	25 (1.08)	nil	1375 (2.51)	606 (1.11)
Years of follow-up, mean	11.77	n/a	15.17	n/a	9.82	n/a	18.09	n/a	18.04	n/a	10.46	n/a	16.55	nil

*AusDiab* Australian Diabetes, Obesity and Lifestyle Study, *BMES* Blue Mountains Eye Study, *CUDS* Crossroads Undiagnosed Diabetes Study, *MCCS* Melbourne Collaborative Cohort Study, *NWAHS* North West Adelaide Health Study, *FPG* Fasting plasma glucose, *SEIFA* Socioeconomic index for areas, *eGFR* estimated glomerular filtration rate, *BMI* body mass index

* Participants who did not fast were recorded as missing FPG

### Cardiovascular disease mortality outcome

The primary endpoint for the CVD risk equation was death from cardiovascular causes, defined as a composite of coronary heart disease (ICD-10 I20-I25) and cerebrovascular disease (ICD-10 I60-I69). A general lack of availability of non-fatal CVD events precluded analysis of a total (fatal plus non-fatal) CVD outcome. CVD mortality was derived from linkage [8] to the National Death Index by the Australian Institute of Health and Welfare. All cohorts were matched collectively with all records successfully linked. Participants were followed until death, or until the censoring date (the last day of follow-up for each participant) - whichever came first.

### Cardiovascular disease risk factors

We collected data on baseline age (years), sex, TC (mmol/L), HDLC (mmol/L), SBP (mm Hg), smoking status, diabetes status, body mass index (BMI; kg/m^2^), SEIFA, educational attainment, estimated glomerular filtration rate (eGFR; ml/min/m^2^), urinary albumin to creatinine ratio (ACR), and family history of CVD. However, ACR was omitted from predictive risk modelling because it was only measured in one study, and family history was omitted because it was inconsistently collected across studies (e.g. self-reported cause of death for mother or father; mother, father, sister, or brother having experienced a CVD event (with no upper age limit); mother, father, sister, or brother having experienced a CHD event prior to age 60 years). TC, HDLC and SBP were measured using standard procedures. Smoking status was dichotomised as current or not current smoking. Diabetes status was defined as a fasting plasma glucose (FPG) ≥126 mg/dl, where available. When data on FPG were missing (Table 1) we used self-reported diabetes status. Participants who were missing FPG and self-reported as not having diabetes were recorded as no diabetes. eGFR was estimated using an enzymatic creatinine assay according to the CKD-EPI equation [8]. The SEIFA score was categorized by national fifths, indexed as 1–5. BMI was derived with objectively measured height and weight. Educational attainment was dichotomised as completed high school or not.

### Statistical methods

Participants were included in the analysis if they were between 40 and 74 years of age and free of CVD at baseline. All continuous variables were tested for log-linear associations with the risk of CVD mortality by graphical means. The only violation found was for eGFR, which had a curvilinear association. To reduce the chance of bias from missing data, multiple imputation by chained equations with 30 imputations was used [9]. Covariates included in our imputation models were baseline age, sex, SBP, TC, HDLC, SEIFA fifth, BMI, eGFR, eGFR^2^, family history of CVD, diabetes status, smoking status, highest level of education and follow-up data on CVD mortality, mortality from any cause and days to censoring or death. As we decided, a priori, that age and sex were likely to be effect modifiers for other risk factors, the imputation model was stratified by sex and by age (in thirds). Analyses were conducted on the complete pooled data set.

Cox proportional hazards regression models were used to quantify the associations between baseline factors and the risk of CVD mortality. When estimating CVD mortality all other causes of death were ignored. The proportional hazards assumption was tested for all covariates included in the model using the Schoenfeld’s global test and by graphical inspection of a plot of the scaled Schoenfeld residuals on a function of time. As an initial exploratory analysis, a model was fitted with only traditional risk factors: age, sex, SBP, TC, HDLC, diabetes and smoking. For the primary prediction model all the exposure variables available were considered as potential prognostic factors, together with all interactions between sex and other variables and between age and all other variables. For the primary prediction model all significant (*p* < 0.05) predictors (risk factors with sex or age interaction terms) in multiple adjusted models were included. We additionally constructed, in an identical way, a low information model, which omitted all clinical variables collected via blood tests, for potential use in non-clinical settings.

From general theory [9, 10], the 5-year risk prediction from a Cox model is approximated as:

$$ \widehat{p}=1 - S{\left(5,\overline{x}\right)}^{\exp (w)} $$

where $S\left(5,\overline{x}\right)$ is the probability of survival (without a CVD death) for a 5-year period for the average person (someone with mean values of each risk factor) at baseline (the start of the 5-year period) in the sample data. Also,

$$ w = {\displaystyle \sum }{b}_1\left({x}_1-{\overline{x}}_1\right)+{b}_2\left({x}_2-{\overline{x}}_2\right) + {b}_3\left({x}_3-{\overline{x}}_3\right) + \dots \dots $$

where the {*x*} are the values taken by any given individual for the risk factors included in the model, the $\left\{\overline{x}\right\}$ are their mean values (in the sample data) and the {*b*} are the regression coefficients (log hazard ratios) from the Cox model.

To obtain a primary risk score, using only sample data, $S\left(5,\overline{x}\right)$ was taken as the mean value after fitting the Cox model for the primary risk model in each of the 30 imputations. Similarly, *w* was taken as the mean over the 30 imputations, but with the {*b*} values taken from the multiple imputation process (thus fixed at each iteration).

### Recalibration

This primary risk score, obtained from the pooled Australian data, may be poorly calibrated for current national purposes for at least two reasons. First, the sample used in each study may be healthier than ‘the average’ at the time of sampling because of the voluntary nature of study participation or the exclusion of subjects who are hard to recruit. Second, because there has been a considerable annual decrease in ‘background’ CVD mortality rates in Australia since the studies used to create the primary score were inaugurated (Additional file 1: Figure S1). The primary score was thus recalibrated [10] using the most current (2013) national data on mortality [11] and risk factors [12], using similar methodology to the GLOBORISK project [13] and an earlier, unadopted, Australian risk score [14] that was recalibrated from European Systematic COronary Risk Evaluation (SCORE) estimates of risk [15].

In our recalibrated score we replaced, for each 5-year age/sex group, $S\left(5,\overline{x}\right)$ with the estimated national 5-year death rate for Australians in 2016 based on the most recent national death statistics, which gives annual CVD mortality rates by 5-year age/sex group, up to 2013 [11]. Also, we replaced $\left\{\overline{x}\right\}$ by the mean values from the most recent (2011/3) comprehensive national health survey [12], obtained by request from the Australian Bureau of Statistics. See Additional file 2: Table S1 for a comparison between Australian national data and the pooled cohorts. Using these sources of data incurs a minor error due to their inclusion of those with prevalent CVD (6.9% in the six datasets used in this paper).

Single-year mortality projections for 2016 were derived from fitting Poisson regression models to 5 year age/sex-specific annual data, for ages 40–79 years, from 2000 to 2013. This model provided a good fit to the data (Additional file 3: Figure S2). Using standard lifetable (‘compound interest’) methods these projections were used to obtain estimated 5-year risks for each 5-year age/sex group, for someone aged at the mid-range of the particular age group. Transition to the next highest age group after 2.5 years was accounted for by taking the single-year estimate of risk as the geometric mean of the estimates in year three of follow-up in the original and next age groups, stratified by sex. Similarly, the value of an individual’s age was rounded to the mid-range of her or his specific 5-year age group when evaluating the *w* component of the recalibrated risk score in each five-year age-group.

### Evaluating the scores

We tested the discrimination of the primary risk score by evaluating its performance in the multiple imputation model using Harrell’s c-statistic [9]. Additionally, we found the corresponding c-statistic in each of the 30 imputation sets and obtained a pooled estimate from a fixed effect meta-analysis [9]. We also compared the discrimination of the primary, low information and traditional risk factor models. Finally, we evaluated discrimination in an external dataset: the Scottish Heart Health Extended Cohort Study [4], approximating SEIFA fifths with the postcode-based deprivation fifths in this study. Although the calibration of the primary risk score does not require evaluation, given that recalibration has been performed, nevertheless it was useful to check that the primary risk score is well calibrated within the sample data. To do such a test, a calibration plot [9] was constructed for a pre-specified arbitrary imputation set (i.e. the sample data from the combined six Australian cohorts with missing data ‘filled-in’) – the first set generated. In addition, the Hosmer-Lemeshow test for survival data [9] was applied to the equal tenths of predicted risk. For comparison with existing scores for CVD mortality, calibration plots were also produced, applied to the same imputation set, for the SCORE models for low- and high-risk European populations [15]. The published 10-year risks from SCORE were transformed to 5-year risks using ‘compound interest’ calculations.

Although external validation would be ideal [10], there is no meaningful way to validate the final, recalibrated model as, by definition, this is a projection into an unknown future Australia. Alternatively, we compared the primary and recalibrated models with each other and with the two SCORE predictions. We computed the estimates for all four algorithms for a woman and a man who did or did not smoke, had or did not have diabetes and had average values of all the other risk factors according to the Australian risk factor survey [12].

Analyses were undertaken using SAS and STATA software; a p value of 0.05 or less was considered significant. All analyses and reporting of the prediction model development and validation were conducted in accordance with the Transparent Reporting of a multivariable prediction model for Individual Prognosis Or Diagnosis (TRIPOD) guidelines.

## Results

Baseline data were collected between the years 1989 and 2003: 54,829 participants (59% women; mean age 56 years) contributed data to the analyses (Table 1). Over a mean follow-up of 16.6 years, 1,375 participants were known to have died from CVD.

The *p*-value for non-proportionality was >0.05 for all covariates, except for smoking where proportional hazards violation was evident (*p* = 0.001), which is explainable by chance, especially as visual inspection of the scaled Schoenfeld residuals showed these to be very minor non-proportional effects. In our exploratory model, with only main effects of traditional risk factors: all these factors were independently predictive (*p* < 0.05) of CVD mortality, with increasing age, TC and SBP, diabetes and smoking associated with an increased risk of CVD mortality, and female sex and increasing HDLC associated with a reduced risk (Table 2), as expected.

**Table 2:** Cox regression coefficients (95% confidence intervals) associated with CVD mortality for each risk factor included in the five-year CVD mortality risk prediction algorithms

Variable (reference group/units)	Primary model	Low information model	Traditional risk factor model
Age	0.373 (0.307, 0.438)	0.310 (0.242, 0.379)	0.168 (0.158, 0.178)
Sex (men)	−1.066 (−1.659, −0.473)	−2.438 (−3.773, −1.103)	−0.535 (−0.655, −0.412)
Systolic blood pressure (10 mmHg)	0.941 (0.653, 1.230)	0.790 (0.490, 1.090)	0.114 (0.088, 0.140)
Total serum cholesterol (mmol/L)	0.122 (0.071, 0.172)		0.140 (0.091, 0.188)
HDL-cholesterol (mmol/L)	−0.482 (−0.794, −0.170)		−0.238 (−0.433, −0.043)
Diabetes (no diabetes)	0.071 (−0.174, 0.316)	0.437 (0.256, 0.617)	0.443 (0.260, 0.625)
Smoker (not current)	2.903 (1.549, 4.257)	3.944 (2.530, 5.358)	0.768 (0.628, 0.907)
SEIFA fifth (most disadvantaged)	−0.119 (−0.174, −0.064)	−0.095 (−0.148, −0.043)	
eGFR (ml/min/m^2^)	−0.127 (−0.165, −0.088)		
eGFR^2^ (ml/min/m^2^)^2^	0.0009 (0.0007, 0.0012)		
Interactions			
sex X diabetes	0.424 (0.041, 0.807)		
sex X SEIFA	−0.115 (−0.198, −0.032)	−0.135 (−0.215, −0.055)	
sex X HDL-cholesterol	0.504 (0.114, 0.895)		
age X systolic blood pressure	−0.013 (−0.017, −0.008)	−0.010 (−0.015, −0.006)	
age X smoker	−0.035 (−0.056, −0.014)	−0.050 (−0.072, −0.028)	
age X sex		0.034 (0.014, 0.054)	

**Fig. 1:**
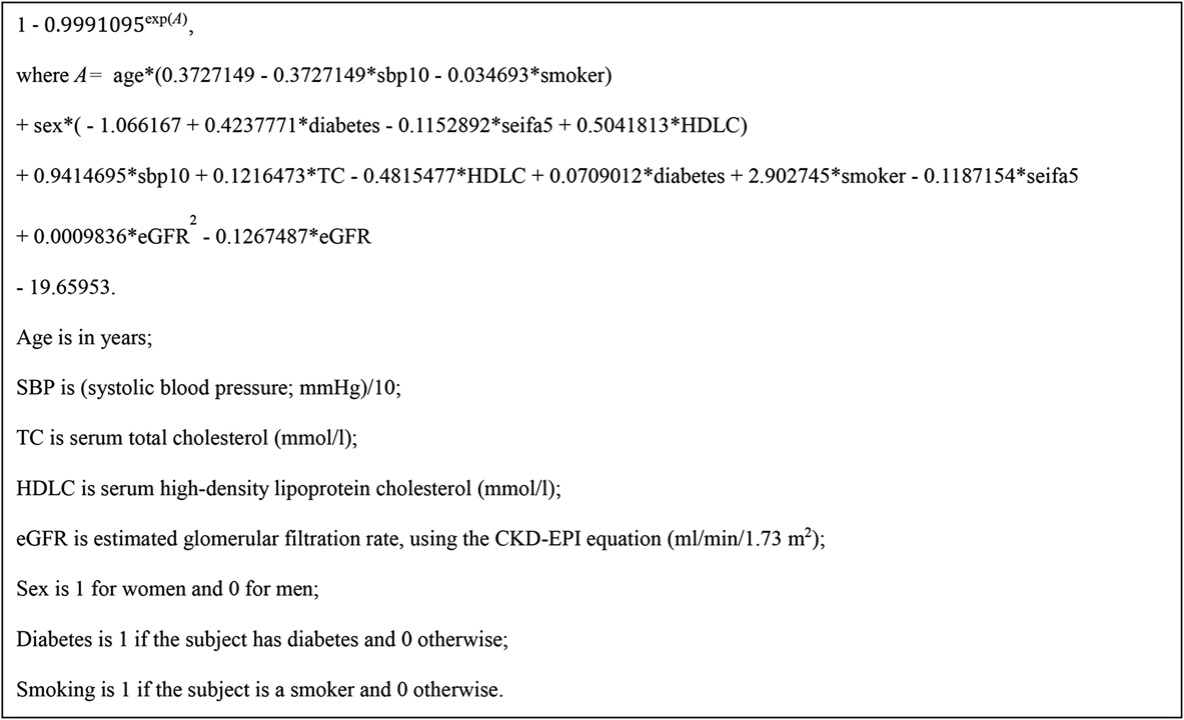
The primary five-year risk score (probability of death from CVD within 5 years)

Taking all the risk factors considered: age, sex, SBP (and its interaction with age), TC, HDLC (and interaction with sex), diabetes (and interaction with sex), smoking (and interaction with age), SEIFA (and interaction with sex) and eGFR and its square and were found to be independently predictive of CVD mortality. The resultant primary risk model, calibrated to the sample data after multiple imputation, is specified in Fig. 1.

In our low information model: age, sex, SBP, diabetes, smoking and SEIFA, plus interactions between sex and SEIFA, age and both SBP and smoking and between age and sex independently predicted CVD mortality.

### Evaluation of the primary risk score

**Fig. 2:**
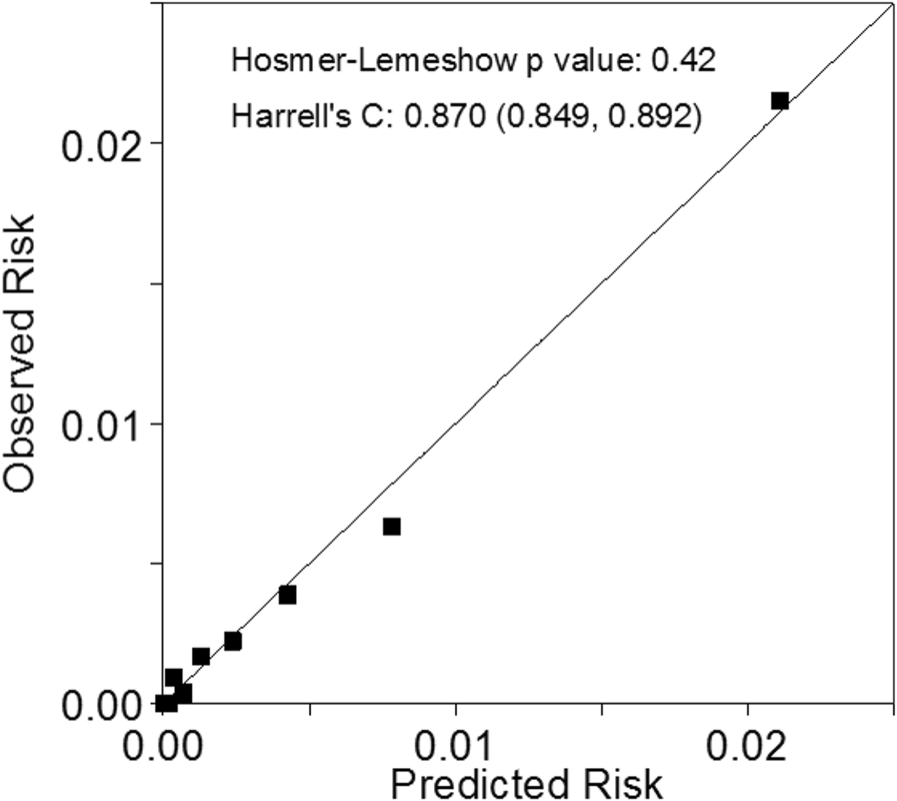
Calibration plot for the primary risk score model (based on sample data) applied to an arbitrary imputed dataset. Predicted risks were categorised into their tenths and observed risks computed within each of these tenths

Internal discrimination of the primary risk score was excellent from the imputed primary model: the c-statistic (95% confidence interval) was 0.910 (0.893, 0.926). When the primary risk score was tested in each individual imputed data set (Additional file 2: Table S2) the pooled c-statistic was 0.871 (0.867, 0.875). As expected, in an arbitrarily chosen single imputed dataset the calibration was good, although risks were heavily clustered at low levels (Fig. 2). The c-statistics for the low information and traditional risk factor scores were 0.836 (0.812, 0.860) and 0.832 (0.807, 0.857), respectively; the p values for a difference from the primary model were both <0.0001. Applied to the Scottish study population, the c-statistic for the primary model was attenuated to 0.751 (0.709, 0.793), so that the score still discriminates well in this external setting.

The European SCORE project’s risk scores for low- and high-risk populations were poorly calibrated to the nominal Australian population in the arbitrary imputed data set illustrated in Fig. 2: even the low-risk score over-predicts risk badly (Additional file 4: Figure S3).

### Recalibration

The recalibrated risk predictions were always higher than those from the primary model, suggesting that the sample populations were generally healthier than typical, contemporary, Australians (Additional file 2: Table S1 and S3). The only exception to this was for the 70–74 year age group where risks were lower from the recalibrated model compared to the primary model, perhaps due to the assumption of a linear relationship between age and CVD mortality risk or due to random error. Risk scores from the recalibrated model, however, produced generally lower predicted risks compared to those from SCORE, both for low- and high-risk populations, suggesting that the attenuation of ‘background’ risk over time has been accounted for by recalibration, at least in a general sense. The only exception was for low-risk subjects (predominantly women) for whom the recalibrated score gave the highest risk predictions. Recalibrated values for the ‘average man’ were three times as large as the primary values, but half the low-risk SCORE values and about a third of the high-risk SCORE values (Table 3). Similar results were observed for the ‘average woman’. Smoking, ageing and being of the male sex increased risk in all scores. Diabetes increased risk in the two Australian scores, but was not accounted for in SCORE, and had a greater impact for women.

**Table 3:** Predicted five-year risks per thousand for ‘average’ men and women, who do/do not smoke or have diabetes, according to the primary and recalibrated Australian risk scores and the SCORE results for low- and high-risk European populations

5-year risk/1000						
	Smoking	Diabetes^a^	Primary	Recalibrated	SCORE low	SCORE high
Men						
	No	No	1.68	4.95	10.32	19.21
	No	Yes	1.81	5.31	10.32	19.21
	Yes	No	4.17	12.23	20.44	38.22
	Yes	Yes	4.48	13.12	20.44	38.22
Women						
	No	No	0.77	1.80	4.31	6.53
	No	Yes	1.26	2.94	4.31	6.53
	Yes	No	1.90	4.45	8.41	12.85
	Yes	Yes	3.11	7.28	8.41	12.85

Risks are for subjects at mean values of continuous risk factors in the 2011–13 Australian Health Survey [12], obtained from the Australian Bureau of Statistics: age 55–59 years, systolic blood pressure = 131.8 mmHg, total cholesterol = 5.25 mmol/l, HDL-cholesterol = 1.24 mmol/l, eGFR = 85.0 (ml/min/m^2^), eGFR squared = 7197 (ml/min/m^2^)^2^ and SEIFA fifth = 3.01985

SCORE values are computed from published 10-year risks [15] using ‘compound interest’ logic. SCORE only takes account of age, sex, systolic blood pressure, total cholesterol and smoking

^a^ SCORE does not include diabetes as a risk factor. The user instructions [15] say that those with diabetes are ‘at very high risk’ which presumably means their predicted 5-year risk is at least 30 per thousand

A proof-of-concept spreadsheet calculator for the recalibrated score was developed in Excel. Additional file 5: Figure S4 is a screen shot from this. A user-friendly ‘publication’ version is under consideration.

## Discussion

Our novel, nationally-recalibrated, risk algorithm includes traditional CVD risk factors, as used in Framingham [2] and the European SCORE [14], in addition to measures of socioeconomic deprivation and chronic kidney disease (eGFR), both of which have been shown to independently predict CVD risk [4, 5, 16]. Accounting for deprivation in a risk algorithm will ensure preventive treatments are more fairly and efficiently allocated and will help to reduce socioeconomic inequalities in CVD.

Although our risk algorithm was derived from a large pool of Australian adults with extensive data on traditional and non-traditional CVD risk factors, we found considerable limitations due to the a priori decision to restrict our analyses to the existing Australian data available to us. Unfortunately the studies we used lacked consistent data on family history of CVD and sufficient data on ACR, both of which have been shown to be independent risk factors of CVD [4, 5, 16]. We would also have liked to take account of Aboriginal and Torres Strait Islander status, but the appropriate data were lacking – future work in this area is needed. Furthermore, missing values were common in the variables we did utilise, rising to as high as 80% for eGFR, which was completely missing in three studies, and 60% for HDLC. We dealt with this issue through cross-study multiple imputation, even though the missing value mechanism varied between studies. This may have introduced some unidentifiable bias. We took the pragmatic approach of assuming that the relationships between variables were consistent across studies. Without this assumption we considered that a principled approach to multiple imputation would not have been possible, without ignoring the bulk of our data. The clustering of missingness within studies has contributed towards the similarity of the estimates across imputations, seen in Additional file 2: Table S2. Imputation may also have increased random error, compared to a complete case analysis [9]. This would tend to have increased p values and so reduced the chance of selection when pruning variables for final models. We also found a price to be paid for data pooling in that the choice of parametric models is limited by the lack of consistent inclusion criteria between studies. Specifically, we preferred to use splines to model the non-linearity of eGFR, but in our age-stratified imputations this proved impossible due to insufficient ‘real’ data across the age spectrum, which caused intractable computational problems. Use of squared eGFR was an approximate compromise.

The endpoint of fatal CVD events is likely to have only detected approximately one third of all CVD events [13]. The original plan was to include all CVD events in the risk algorithm, but we were unable to do so due to insufficient data on non-fatal CVD events being available to us. This limits the utility of our derived risk score in Australian clinical practice, although the European Society of Cardiology does promote the use of a fatal CVD risk score [15].

## Conclusions

We have the developed a CVD risk score based on purely Australian cohort data. This has the advantage of being locally relevant, but has three disadvantages. One, the risk experience of the subjects in the datasets occurred in the past, and ‘background’ risk has lessened over the years. Two, recruitment to the six cohorts analysed was evidently not entirely at random; for example those unable to be contacted were omitted – the ‘healthy cohort’ effect. Virtually all previous CVD risk scores have had these same two problems; here the problems were addressed by using recalibration to contemporary national statistics. The third problem is that the data are not ideal for the purpose: data on relevant risk factors are often missing, sometimes in whole cohorts, and data on non-fatal CVD events were essentially completely missing. Except in approximate ways, as enacted here, this problem is not solvable with Australian data, in so far as we could ascertain. Nevertheless, the new risk score represents an innovative approach to predicting 5-year CVD mortality risk for the Australian population that makes good use of the locally available data, and the methodology we have developed could be used outside Australia by recalibrating our primary risk score to local conditions using appropriate national statistical data. Implementation of our Australian CVD mortality risk prediction tool would be expected to lead to better prediction of true risk than is currently available.

## Additional files

Additional file 1:
**Figure S1.** Cardiovascular disease mortality rates, 2000-2013, in Australian men (solid lines) and women (dashed lines) in two illustrative age groups: 60-64 years (light lines) and 70-74 years (dark lines). A logarithmic vertical scale is used. Note: Source of raw data: Australian Bureau of Statistics [17]. (TIF 142 kb)
Additional file 2:
**Table S1.** CVD mortality rate and risk factor means (or percentages) for the National Health Survey (NHS) and the pooled cohort used for risk score development, by sex and age group (years). **Table S2.** Mean values of each prognostic factor and other key statistics in each imputed dataset. Note: The constant term takes the place of 19.65953 in the equation for the primary risk score (in Fig. 1). **Table S3.** Predicted five-year risks per thousand by age group for ‘average’ men and women, who do/do not smoke or have diabetes, according to the primary and recalibrated Australian risk scores and the SCORE results for low- and high-risk European populations. Risks are for subjects at mean values of continuous risk factors in the 2011–13 Australian Health Survey [9], obtained from the Australian Bureau of Statistics: systolic blood pressure = 131.8 mmHg, total cholesterol = 5.25 mmol/l, HDL-cholesterol = 1.24 mmol/l, eGFR = 85.0, eGFR squared = 7197 and SEIFA fifth = 3.02. SCORE values computed from published 10-year risks [12] using 'compound interest' logic. SCORE takes account of age, sex, systolic blood pressure, total cholesterol and smoking.^1^SCORE does not include diabetes as a risk factor. The user instructions [12] say that those with diabetes are 'at very high risk' which presumably means their predicted 5-year risk is at least 30 per thousand. (DOCX 30 kb)
Additional file 3:
**Figure S2.** Fitted versus observed annual age/sex specific cardiovascular death rates, Australia 2000-2013. Fitted values derive from a Poisson regression model. Dots are for men and pluses for women. (TIF 133 kb)
Additional file 4:
**Figure S3.** Calibration plots for SCORE models applied to an arbitrary imputed dataset (the same one as in Fig. 2). Predicted five-year risks were categorised into their tenths and observed risks computed within each of these tenths. A: the European SCORE model for low-risk populations; B: the European SCORE model for high-risk populations. (TIF 267 kb)
Additional file 5
**Figure S4.** Screen shot from working version of the CVD risk prediction tool. (TIF 392 kb)

## Abbreviations

ACR: Urinary albumin to creatinine ratio
AIHW: Australian institute of health and welfare
AusDiab: Australian diabetes, obesity and lifestyle study
BMES: Blue mountains eye study
BMI: Body mass index
CUDS: Crossroads undiagnosed diabetes study
CVD: Cardiovascular disease
eGFR: Estimated glomerular filtration rate
FPG: Fasting plasma glucose
HDLC: High-density lipoprotein cholesterol
HREC: Human research ethics committee
MCCS: Melbourne collaborative cohort study
NWAHS: North west adelaide health
SBP: Systolic blood pressure
SCORE: Systematic coronary risk evaluation
SEIFA: Socioeconomic index for areas
TC: Total cholesterol
TRIPOD: Transparent reporting of a multivariable prediction model for individual prognosis or diagnosis
