# Supplementary figures and images for: Development of an Australian cardiovascular disease mortality risk score using multiple imputation and recalibration from national statistics
Source: BMC Cardiovasc Disord. 2017 Jan 6;17:17. doi: 10.1186/s12872-016-0462-5 (PMC5219754; doi:10.1186/s12872-016-0462-5)

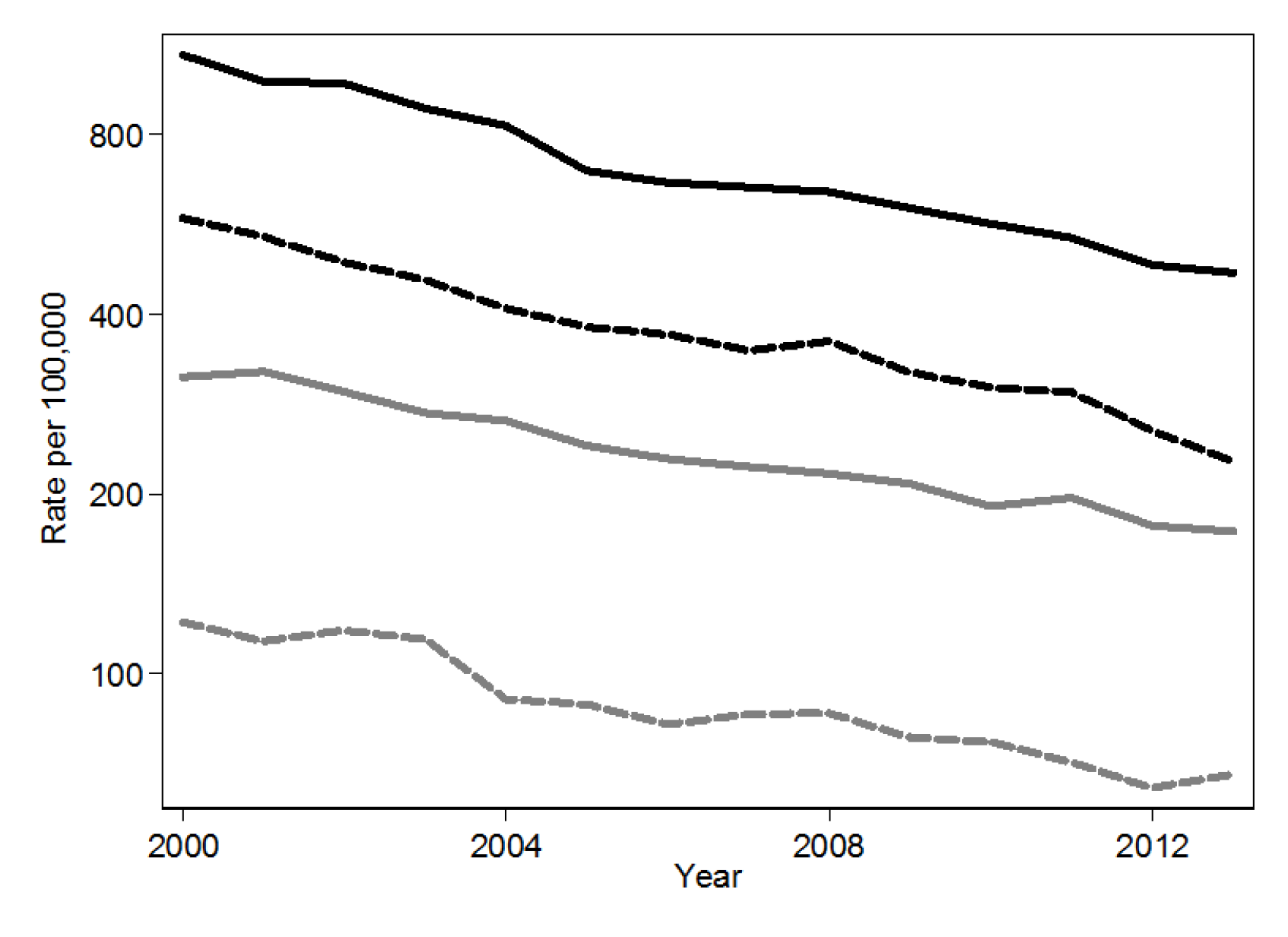

Supplement: Additional file 1: — Figure S1. Cardiovascular disease mortality rates, 2000-2013, in Australian men (solid lines) and women (dashed lines) in two illustrative age groups: 60-64 years (light lines) and 70-74 years (dark lines). A logarithmic vertical scale is used. Note: Source of raw data: Australian Bureau of Statistics [17]. (TIF 142 kb) [file 12872_2016_462_MOESM1_ESM.tif]

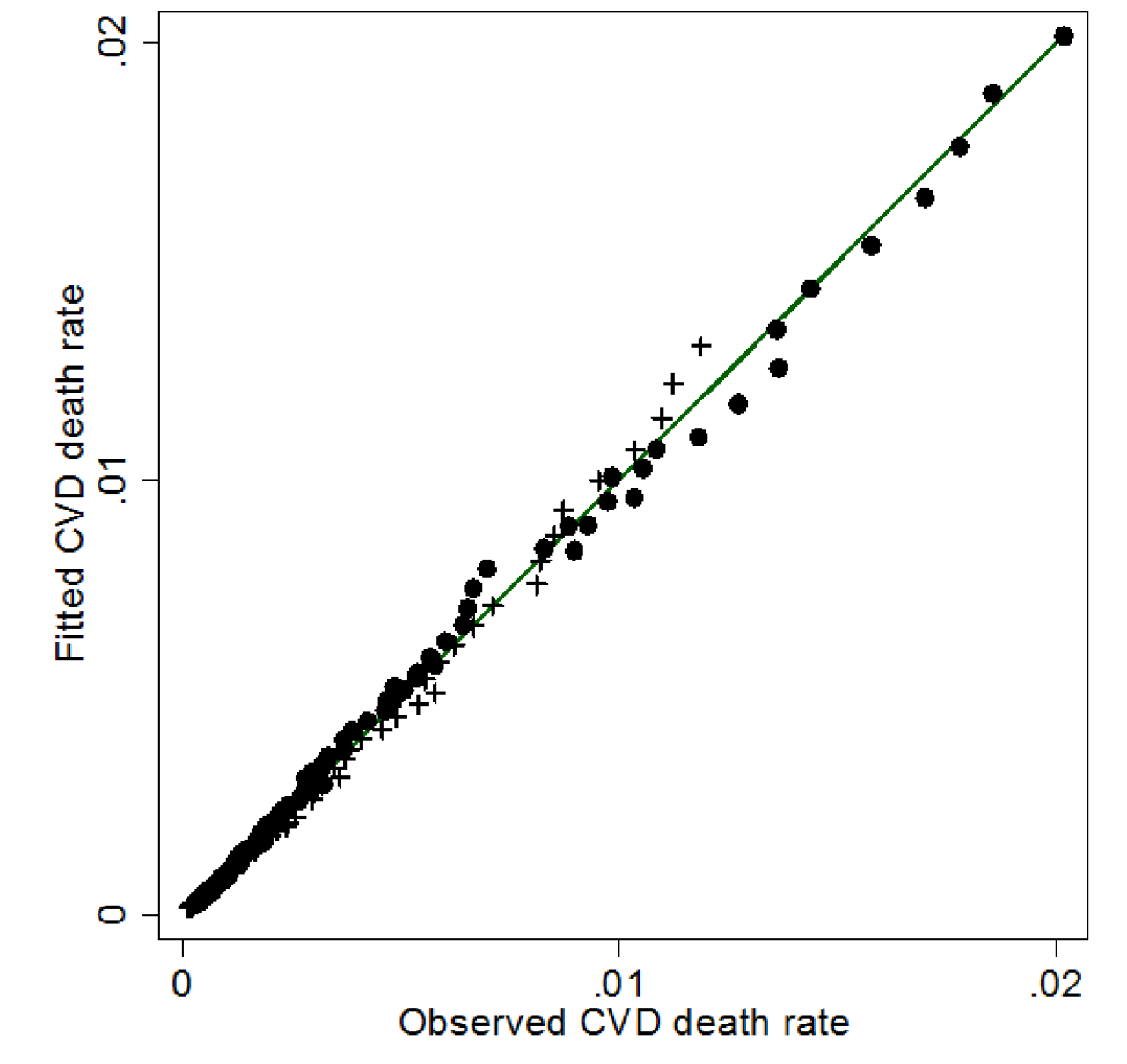

Supplement: Additional file 3: — Figure S2. Fitted versus observed annual age/sex specific cardiovascular death rates, Australia 2000-2013. Fitted values derive from a Poisson regression model. Dots are for men and pluses for women. (TIF 133 kb) [file 12872_2016_462_MOESM3_ESM.tif]

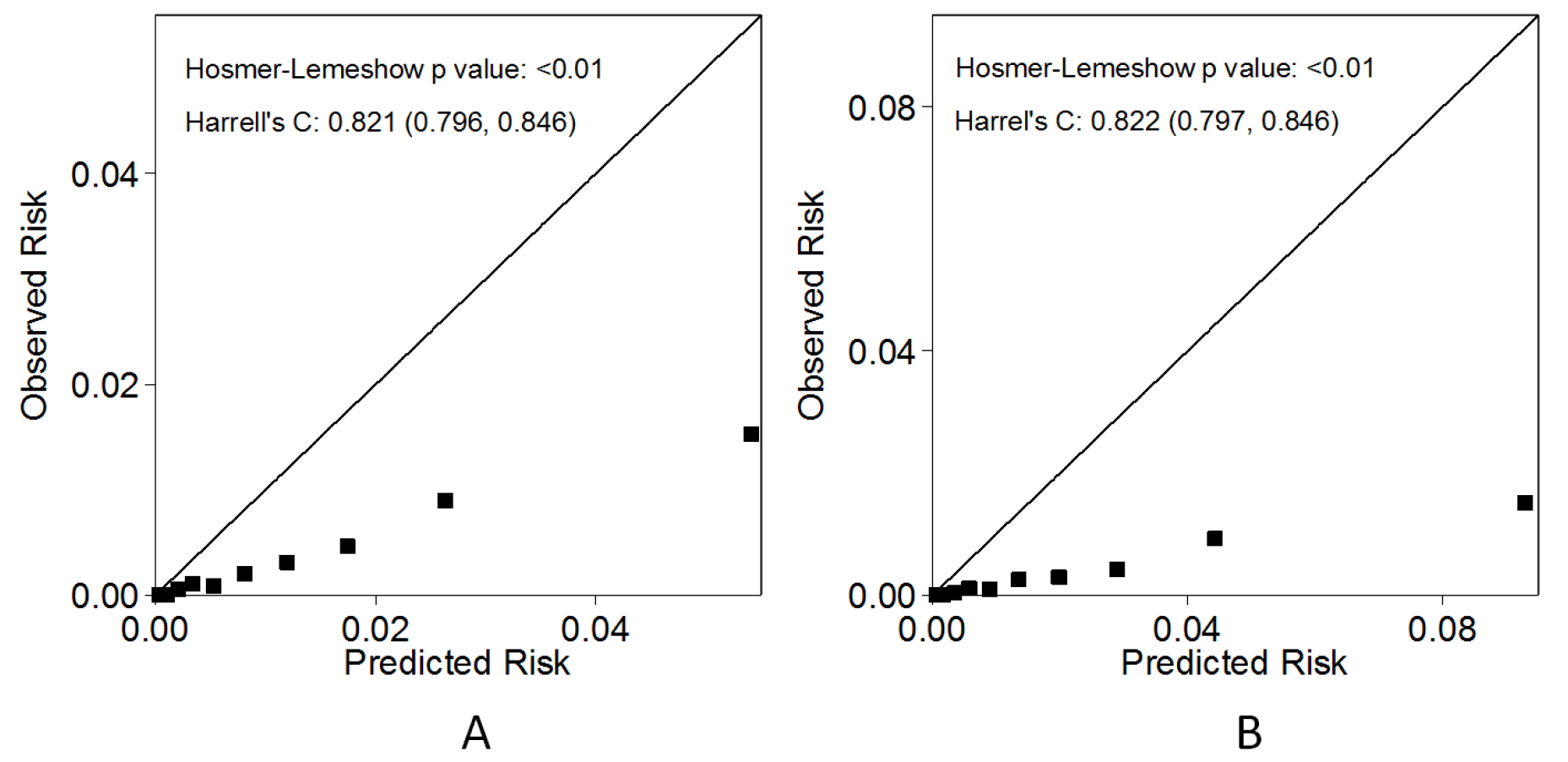

Supplement: Additional file 4: — Figure S3. Calibration plots for SCORE models applied to an arbitrary imputed dataset (the same one as in Fig. 2). Predicted five-year risks were categorised into their tenths and observed risks computed within each of these tenths. A: the European SCORE model for low-risk populations; B: the European SCORE model for high-risk populations. (TIF 267 kb) [file 12872_2016_462_MOESM4_ESM.tif]

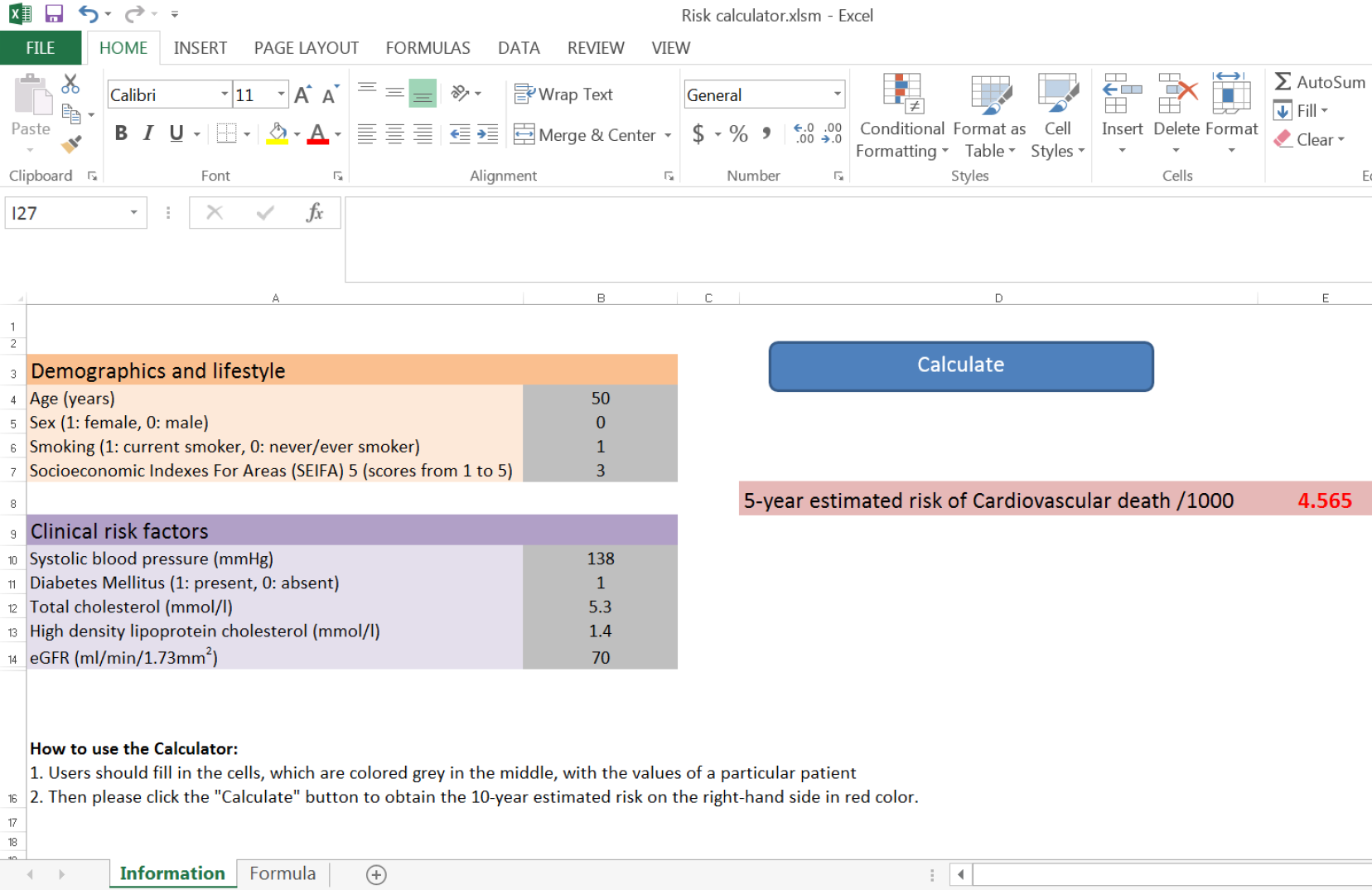

Supplement: Additional file 5 — Figure S4. Screen shot from working version of the CVD risk prediction tool. (TIF 392 kb) [file 12872_2016_462_MOESM5_ESM.tif]
