# Supplementary material for: Development of an Australian cardiovascular disease mortality risk score using multiple imputation and recalibration from national statistics
Source: BMC Cardiovasc Disord. 2017 Jan 6;17:17. doi: 10.1186/s12872-016-0462-5 (PMC5219754; doi:10.1186/s12872-016-0462-5)
Supplement: Additional file 2: — Table S1. CVD mortality rate and risk factor means (or percentages) for the National Health Survey (NHS) and the pooled cohort used for risk score development, by sex and age group (years). Table S2. Mean values of each prognostic factor and other key statistics in each imputed dataset. Note: The constant term takes the place of 19.65953 in the equation for the primary risk score (in Fig. 1). Table S3. Predicted five-year risks per thousand by age group for ‘average’ men and women, who do/do not smoke or have diabetes, according to the primary and recalibrated Australian risk scores and the SCORE results for low- and high-risk European populations. Risks are for subjects at mean values of continuous risk factors in the 2011–13 Australian Health Survey [9], obtained from the Australian Bureau of Statistics: systolic blood pressure = 131.8 mmHg, total cholesterol = 5.25 mmol/l, HDL-cholesterol = 1.24 mmol/l, eGFR = 85.0, eGFR squared = 7197 and SEIFA fifth = 3.02. SCORE values computed from published 10-year risks [12] using 'compound interest' logic. SCORE takes account of age, sex, systolic blood pressure, total cholesterol and smoking.1SCORE does not include diabetes as a risk factor. The user instructions [12] say that those with diabetes are 'at very high risk' which presumably means their predicted 5-year risk is at least 30 per thousand. (DOCX 30 kb) [file 12872_2016_462_MOESM2_ESM.docx]

**Supplementary Table 1**

|  | 40-44 | | 45-49 | | 50-54 | | 55-59 | | 60-64 | | 65-69 | | 70-74 | |
| --- | --- | --- | --- | --- | --- | --- | --- | --- | --- | --- | --- | --- | --- | --- |
|  | NHS | Cohort | NHS | Cohort | NHS | Cohort | NHS | Cohort | NHS | Cohort | NHS | Cohort | NHS | Cohort |
| **Men** |  | | | | | | | | | | | | | |
| CVD mortality rate (per 100,000) | 27 | 20 | 50 | 29 | 76 | 57 | 114 | 131 | 173 | 304 | 295 | 591 | 467 | 1161 |
| SBP (mmHg) | 121.6 | 125.2 | 124.0 | 128.5 | 128.1 | 132.4 | 131.8 | 136.2 | 132.4 | 140.1 | 132.4 | 143.8 | 137.2 | 146.9 |
| TC (mmol/L) | 5.3 | 5.7 | 5.5 | 5.8 | 5.3 | 5.7 | 5.2 | 5.7 | 5.1 | 5.8 | 4.9 | 5.7 | 4.8 | 5.6 |
| HDL-C (mmol/L) | 1.2 | 1.2 | 1.2 | 1.2 | 1.2 | 1.2 | 1.2 | 1.2 | 1.2 | 1.3 | 1.2 | 1.3 | 1.2 | 1.3 |
| SEIFA fifth | 3.2 | 2.8 | 3.2 | 2.9 | 3.1 | 3.0 | 3.0 | 2.8 | 3.1 | 2.4 | 2.9 | 2.4 | 2.9 | 2.4 |
| eGFR (ml/min/m^2^) | 88.9 | 102.5 | 87.1 | 97.7 | 86.4 | 90.3 | 85.0 | 85.9 | 83.7 | 80.1 | 79.2 | 74.8 | 75.4 | 70.5 |
| Current smoker (%) | 23.6 | 25.3 | 23.9 | 21.3 | 24.1 | 19.3 | 18.2 | 19.3 | 14.5 | 17.4 | 13.4 | 13.0 | 8.4 | 12.8 |
| Diabetes (%) | 4.7 | 3.5 | 6.2 | 4.2 | 7.1 | 6.4 | 8.9 | 10.0 | 12.3 | 10.2 | 16.9 | 11.3 | 23.8 | 11.1 |
|  |  | | | | | | | | | | | | | |
| **Women** |  | | | | | | | | | | | | | |
| CVD mortality rate (per 100,000) | 10 | 3 | 19 | 6 | 26 | 19 | 40 | 45 | 68 | 167 | 114 | 359 | 226 | 956 |
| SBP (mmHg) | 114.8 | 116.4 | 119.6 | 121.5 | 122.4 | 129.6 | 126.8 | 133.6 | 129.3 | 139.1 | 134.1 | 143.4 | 137.1 | 147.7 |
| TC (mmol/L) | 5.0 | 5.3 | 5.3 | 5.5 | 5.5 | 5.8 | 5.7 | 6.0 | 5.5 | 6.2 | 5.4 | 6.2 | 5.2 | 6.3 |
| HDL-C (mmol/L) | 1.5 | 1.5 | 1.5 | 1.6 | 1.5 | 1.6 | 1.5 | 1.6 | 1.5 | 1.5 | 1.5 | 1.5 | 1.5 | 1.5 |
| SEIFA fifth | 3.1 | 2.9 | 3.2 | 3.0 | 3.1 | 2.9 | 3.0 | 2.8 | 3.1 | 2.4 | 3.0 | 2.3 | 2.9 | 2.4 |
| eGFR (ml/min/m^2^) | 88.8 | 104.1 | 87.9 | 99.8 | 87.4 | 90.0 | 86.5 | 84.3 | 84.7 | 78.5 | 81.9 | 72.3 | 75.9 | 67.4 |
| Current smoker (%) | 17.3 | 22.1 | 17.4 | 15.9 | 20.4 | 15.4 | 16.2 | 15.4 | 11.2 | 12.4 | 8.2 | 10.4 | 6.6 | 9.1 |
| Diabetes (%) | 3.3 | 1.7 | 2.5 | 3.5 | 2.5 | 3.2 | 4.2 | 4.6 | 6.6 | 5.6 | 6.7 | 6.8 | 13.2 | 7.3 |

**Supplementary Table 2**

| Imputation | Age | Sex  (%) | SBP | TC | HDLC | Diabetes (%) | SEIFA 5th | Smoking (%) | eGFR | eGFR^2^ | Constant | S (5,$\bar{x}$) | c-statistic  (95%CI) |
| --- | --- | --- | --- | --- | --- | --- | --- | --- | --- | --- | --- | --- | --- |
| 1 | 56 | 59 | 136.0171 | 5.615072 | 1.425984 | 4.44 | 3.070018 | 12.63 | 75.29191 | 6056.065 | 19.64088 | 0.999113 | 0.870 (0.849, 0.892) |
| 2 | 56 | 59 | 136.0139 | 5.615118 | 1.426866 | 4.44 | 3.070419 | 12.62 | 76.01022 | 6191.467 | 19.68157 | 0.99908 | 0.868 (0.846, 0.890) |
| 3 | 56 | 59 | 136.0193 | 5.615664 | 1.425094 | 4.44 | 3.070218 | 12.62 | 75.85944 | 6157.853 | 19.66817 | 0.999088 | 0.870 (0.849, 0.892) |
| 4 | 56 | 59 | 136.0194 | 5.616068 | 1.424727 | 4.44 | 3.070164 | 12.62 | 75.86526 | 6168.906 | 19.67782 | 0.999088 | 0.870 (0.849, 0.892) |
| 5 | 56 | 59 | 136.0143 | 5.616265 | 1.424861 | 4.44 | 3.071057 | 12.62 | 75.31089 | 6055.822 | 19.63701 | 0.999104 | 0.869 (0.847, 0.891) |
| 6 | 56 | 59 | 136.0157 | 5.616026 | 1.423401 | 4.44 | 3.070911 | 12.64 | 75.74668 | 6138.713 | 19.66393 | 0.999108 | 0.872 (0.851, 0.893) |
| 7 | 56 | 59 | 136.0159 | 5.615746 | 1.421732 | 4.44 | 3.070145 | 12.62 | 75.35334 | 6062.053 | 19.63849 | 0.99912 | 0.872 (0.850, 0.893) |
| 8 | 56 | 59 | 136.0130 | 5.615412 | 1.422475 | 4.44 | 3.070838 | 12.63 | 75.59434 | 6115.779 | 19.65949 | 0.999127 | 0.872 (0.850, 0.893) |
| 9 | 56 | 59 | 136.0245 | 5.615676 | 1.420222 | 4.44 | 3.071112 | 12.63 | 76.01477 | 6199.119 | 19.69009 | 0.999109 | 0.872 (0.850, 0.893) |
| 10 | 56 | 59 | 136.0217 | 5.616141 | 1.425476 | 4.44 | 3.070711 | 12.62 | 74.99915 | 6013.493 | 19.63617 | 0.999129 | 0.872 (0.851, 0.893) |
| 11 | 56 | 59 | 136.0191 | 5.615732 | 1.420666 | 4.44 | 3.070145 | 12.61 | 75.69675 | 6135.456 | 19.66718 | 0.999114 | 0.872 (0.850, 0.893) |
| 12 | 56 | 59 | 136.0193 | 5.615037 | 1.423811 | 4.44 | 3.070547 | 12.64 | 75.71041 | 6118.701 | 19.64996 | 0.999118 | 0.869 (0.846, 0.891) |
| 13 | 56 | 59 | 136.0116 | 5.615311 | 1.423841 | 4.44 | 3.070930 | 12.64 | 76.04732 | 6188.574 | 19.67536 | 0.999109 | 0.871 (0.850, 0.892) |
| 14 | 56 | 59 | 136.0122 | 5.616838 | 1.416824 | 4.44 | 3.070492 | 12.63 | 75.57672 | 6116.056 | 19.66391 | 0.99909 | 0.870 (0.849, 0.891) |
| 15 | 56 | 59 | 136.0175 | 5.615151 | 1.423522 | 4.44 | 3.070747 | 12.64 | 75.29490 | 6067.404 | 19.65235 | 0.999094 | 0.873 (0.852, 0.894) |
| 16 | 56 | 59 | 136.0144 | 5.615974 | 1.419673 | 4.44 | 3.070364 | 12.64 | 75.64895 | 6122.271 | 19.66146 | 0.999111 | 0.872 (0.851, 0.894) |
| 17 | 56 | 59 | 136.0144 | 5.616295 | 1.423632 | 4.44 | 3.070401 | 12.67 | 75.09438 | 6012.121 | 19.62335 | 0.999121 | 0.868 (0.846, 0.890) |
| 18 | 56 | 59 | 136.0177 | 5.615427 | 1.420013 | 4.44 | 3.069635 | 12.62 | 75.64045 | 6109.875 | 19.64971 | 0.999117 | 0.872 (0.850, 0.893) |
| 19 | 56 | 59 | 136.0151 | 5.616046 | 1.426482 | 4.44 | 3.070620 | 12.63 | 76.21217 | 6203.649 | 19.66889 | 0.999087 | 0.867 (0.845, 0.889) |
| 20 | 56 | 59 | 136.0207 | 5.615652 | 1.424364 | 4.44 | 3.070911 | 12.63 | 75.77891 | 6142.853 | 19.66408 | 0.999121 | 0.869 (0.847, 0.891) |
| 21 | 56 | 59 | 136.0117 | 5.615641 | 1.423280 | 4.44 | 3.071057 | 12.63 | 75.81378 | 6139.536 | 19.65636 | 0.999116 | 0.872 (0.851, 0.893) |
| 22 | 56 | 59 | 136.0191 | 5.616560 | 1.422961 | 4.44 | 3.070200 | 12.62 | 75.37234 | 6073.695 | 19.64778 | 0.999143 | 0.872 (0.851, 0.894) |
| 23 | 56 | 59 | 136.0155 | 5.616444 | 1.422497 | 4.44 | 3.070346 | 12.63 | 75.76440 | 6147.776 | 19.67066 | 0.999139 | 0.872 (0.851, 0.894) |
| 24 | 56 | 59 | 136.0171 | 5.616370 | 1.418647 | 4.44 | 3.070838 | 12.63 | 74.98529 | 6008.234 | 19.63279 | 0.999102 | 0.872 (0.851, 0.894) |
| 25 | 56 | 59 | 136.0154 | 5.615710 | 1.421151 | 4.44 | 3.070474 | 12.65 | 75.35648 | 6061.658 | 19.63797 | 0.999102 | 0.865 (0.843, 0.888) |
| 26 | 56 | 59 | 136.0168 | 5.615950 | 1.426891 | 4.44 | 3.070765 | 12.63 | 75.35648 | 6251.176 | 19.6937 | 0.999102 | 0.872 (0.850, 0.893) |
| 27 | 56 | 59 | 136.0171 | 5.616125 | 1.417436 | 4.44 | 3.070382 | 12.64 | 76.10821 | 6203.964 | 19.68308 | 0.999057 | 0.869 (0.847, 0.891) |
| 28 | 56 | 59 | 136.0143 | 5.616336 | 1.417670 | 4.44 | 3.071076 | 12.65 | 75.63108 | 6124.170 | 19.6646 | 0.999112 | 0.872 (0.850, 0.893) |
| 29 | 56 | 59 | 136.0156 | 5.615334 | 1.423117 | 4.44 | 3.070656 | 12.61 | 75.63108 | 6107.729 | 19.65209 | 0.999136 | 0.852 (0.829, 0.874) |
| 30 | 56 | 59 | 136.0175 | 5.616161 | 1.425870 | 4.44 | 3.070474 | 12.63 | 75.86901 | 6166.875 | 19.67692 | 0.99913 | 0.851 (0.828, 0.874) |

**Supplementary Table 3:**

|  |  | 5-year risk/1000 | | | |
| --- | --- | --- | --- | --- | --- |
|  | Age (years) | Primary | Recalibrated | SCORE low | SCORE high |
| **Non-smoker without diabetes^1^** | | | |  |  |
| Men |  |  |  |  |  |
|  | 40-44 | 0.08 | 1.59 | 1.89 | 3.80 |
|  | 45-49 | 0.22 | 2.23 | 3.60 | 7.04 |
|  | 50-54 | 0.60 | 3.09 | 6.30 | 12.00 |
|  | 55-59 | 1.68 | 4.95 | 10.32 | 19.21 |
|  | 60-64 | 4.69 | 8.64 | 16.06 | 29.27 |
|  | 65-69 | 13.04 | 14.96 | 23.98 | 42.84 |
|  | 70-74 | 35.96 | 26.77 | 34.62 | 60.69 |
| Women |  |  |  |  |  |
|  | 40-44 | 0.04 | 1.36 | 0.43 | 0.69 |
|  | 45-49 | 0.10 | 1.25 | 1.03 | 1.63 |
|  | 50-54 | 0.28 | 1.36 | 2.20 | 3.41 |
|  | 55-59 | 0.77 | 1.80 | 4.31 | 6.53 |
|  | 60-64 | 2.14 | 3.20 | 7.84 | 11.66 |
|  | 65-69 | 5.95 | 6.15 | 13.49 | 19.69 |
|  | 70-74 | 16.52 | 14.11 | 22.10 | 31.75 |
| **Non-smoker with diabetes^1^** | | |  |  |  |
| Men | | |  |  |  |
|  | 40-44 | 0.08 | 1.71 | 1.89 | 3.80 |
|  | 45-49 | 0.23 | 2.39 | 3.60 | 7.04 |
|  | 50-54 | 0.65 | 3.32 | 6.30 | 12.00 |
|  | 55-59 | 1.81 | 5.31 | 10.32 | 19.21 |
|  | 60-64 | 5.04 | 9.27 | 16.06 | 29.27 |
|  | 65-69 | 13.99 | 16.05 | 23.98 | 42.84 |
|  | 70-74 | 38.55 | 28.71 | 34.62 | 60.69 |
| Women |  |  |  |  |  |
|  | 40-44 | 0.06 | 2.22 | 0.43 | 0.69 |
|  | 45-49 | 0.16 | 2.05 | 1.03 | 1.63 |
|  | 50-54 | 0.45 | 2.23 | 2.20 | 3.41 |
|  | 55-59 | 1.26 | 2.94 | 4.31 | 6.53 |
|  | 60-64 | 3.50 | 5.25 | 7.84 | 11.66 |
|  | 65-69 | 9.74 | 10.07 | 13.49 | 19.69 |
|  | 70-74 | 26.95 | 23.03 | 22.10 | 31.75 |
|  |  |  |  |  |  |

|  |  | 5-year risk/1000 | | | |
| --- | --- | --- | --- | --- | --- |
|  | Age (years) | Primary | Recalibrated | SCORE low | SCORE high |
| **Smoker without diabetes^1^** | | |  |  |  |
| Men |  |  |  |  |  |
|  | 40-44 | 0.32 | 6.63 | 3.78 | 7.61 |
|  | 45-49 | 0.76 | 7.79 | 7.17 | 14.06 |
|  | 50-54 | 1.78 | 9.08 | 12.50 | 23.93 |
|  | 55-59 | 4.17 | 12.23 | 20.44 | 38.22 |
|  | 60-64 | 9.76 | 17.91 | 31.75 | 58.11 |
|  | 65-69 | 22.74 | 26.06 | 47.34 | 84.89 |
|  | 70-74 | 52.53 | 39.19 | 68.23 | 120.06 |
| Women |  |  |  |  |  |
|  | 40-44 | 0.15 | 5.65 | 0.83 | 1.37 |
|  | 45-49 | 0.35 | 4.39 | 2.01 | 3.21 |
|  | 50-54 | 0.81 | 4.01 | 4.30 | 6.71 |
|  | 55-59 | 1.90 | 4.45 | 8.41 | 12.85 |
|  | 60-64 | 4.45 | 6.67 | 15.32 | 22.95 |
|  | 65-69 | 10.41 | 10.76 | 26.33 | 38.74 |
|  | 70-74 | 24.25 | 20.72 | 43.14 | 62.44 |

**Smoker with diabetes^1^**

| Men |  |  |  |  |  |
| --- | --- | --- | --- | --- | --- |
|  | 40-44 | 0.35 | 7.11 | 3.78 | 7.61 |
|  | 45-49 | 0.82 | 8.36 | 7.17 | 14.06 |
|  | 50-54 | 1.91 | 9.75 | 12.50 | 23.93 |
|  | 55-59 | 4.48 | 13.12 | 20.44 | 38.22 |
|  | 60-64 | 10.47 | 19.22 | 31.75 | 58.11 |
|  | 65-69 | 24.39 | 27.95 | 47.34 | 84.89 |
|  | 70-74 | 56.28 | 42.01 | 68.23 | 120.06 |
| Women |  |  |  |  |  |
|  | 40-44 | 0.24 | 9.24 | 0.83 | 1.37 |
|  | 45-49 | 0.57 | 7.18 | 2.01 | 3.21 |
|  | 50-54 | 1.33 | 6.57 | 4.30 | 6.71 |
|  | 55-59 | 3.11 | 7.28 | 8.41 | 12.85 |
|  | 60-64 | 7.29 | 10.91 | 15.32 | 22.95 |
|  | 65-69 | 17.01 | 17.58 | 26.33 | 38.74 |
|  | 70-74 | 39.45 | 33.75 | 43.14 | 62.44 |
